# Companion Animals as Potential Reservoirs of Antibiotic Resistant Diarrheagenic *Escherichia coli* in Shandong, China

**DOI:** 10.3390/antibiotics11060828

**Published:** 2022-06-20

**Authors:** Lulu Cui, Xiaonan Zhao, Ruibo Li, Yu Han, Guijuan Hao, Guisheng Wang, Shuhong Sun

**Affiliations:** 1 Shandong Provincial Key Laboratory of Animal Biotechnology and Disease Control and Prevention, College of Animal Science and Veterinary Medicine, Shandong Agricultural University, Tai’an 271018, China; 2021010090@sdau.edu.cn (L.C.); 2020120695@sdau.edu.cn (R.L.); 2021120549@sdau.edu.cn (Y.H.); haogj2020@sdau.edu.cn (G.H.); 2 Institute of Animal Science and Veterinary Medicine, Shandong Academy of Agricultural Sciences, Jinan 250100, China; zhaoxiaonan@shandong.cn; 3 Shandong Animal Disease Prevention and Control Center, Jinan 250100, China

**Keywords:** antimicrobial resistance, *Escherichia coli*, ESBLs, phenotype, companion animals, diarrheagenic

## Abstract

Antibiotic resistance genes of *Escherichia coli* (*E. coli*) from companion animals were still poorly understood. Here, we investigated the extended-spectrum β-lactamases (ESBLs) resistance genes of *E. coli* from companion animals in Shandong, China. A total of 79 isolates (80.6%) were recovered from 98 healthy or diarrheal companion animals in 2021, among which ESBLs-producing isolates accounted for 43.0% (34/79), and more than half of ESBL *E. coli* (ESBL-EC) strains (*n* = 19) were isolated from healthy companion animals. Diarrheagenic *E. coli* isolates (45.6%, *n* = 36) were represented by enterotoxigenic (ETEC) (32.9%), enteropathogenic (EPEC) (10.1%) and enteroinvasive (EIEC) (2.6%), 20 isolates of which were from healthy pets. Among tested antibiotics, resistance to tetracycline (64.6%) was the most commonly observed, followed by doxycycline (59.5%) and ampicillin (53.2%). Notably, all isolates were susceptible to meropenem. The multidrug-resistant (MDR) rate was 49.4%, 20 isolates of which were ESBLs producers; moreover, 23.4%, 16.4% of ESBL-EC strains were resistant to 5 or more, 7 or more antibiotics, respectively. Among the 5 β-lactamase resistance genes, the most frequent gene was *bla*_CTX-M_ (60.76%), followed by *bla*_SHV_ (40.51%). The plasmid-mediated quinolone resistance (PMQR) gene *aac(6’)-Ib-cr* was detected in 35 isolates. Additionally, ESBL-associated genes (i.e., *bla*_CTX-M_, *bla*_SHV_) were found in 76.5% ESBL-EC strains, with six isolates carrying *bla*_CTX-M_ and *bla*_SHV_. The marker gene of high-pathogenicity island gene *irp2* (encoding iron capture systems) was the most frequency virulence gene. Our results showed that ESBL-EC were widespread in healthy or diarrhea companion animals, especially healthy pets, which may be a potential reservoir of antibiotic resistance, therefore, enhancing a risk to public and animal health.

## 1. Introduction

*Escherichia coli* (*E. coli*), a member of the *Enterobacteriaceae* family, widely distributes in healthy humans and animals [1]. Most *E. coli* strains colonize harmlessly in the intestines and rarely cause disease in healthy individuals; however, some pathogenic *E. coli* strains may lead to diarrhea in both humans and companion animals [2,3]. According to the virulence characteristics and clinical symptoms of diarrheagenic *Escherichia coli* (DEC), they have been further divided into five categories [4]: enteroinvasive (EIEC), enterotoxigenic (ETEC), enteropathogenic (EPEC), enterohemorrhagic (Shiga toxin-producing) (STEC), and enteroaggregative (EAEC). EPEC and ETEC strains have been reported from healthy or diarrhea companion animals [2,5,6]. EIEC are studied mainly in human infections [7,8], but studies in companion animals are limited.

The emergence of antimicrobial resistance in bacteria is an ongoing severe public health problem and results in 700,000 deaths annually [9]. Antibiotic misuse has led to the increasing emergence of multidrug-resistant (MDR) and pan-resistant strains in humans, companion animals, water and food animals [10]. Companion animals are kept for company, entertainment, or compassion for humans. In China, 55.03 million dogs and 44.12 million cats were kept in cities in 2019. The close contact between companion animals and humans enhances the risk of bacterial and even antimicrobial-resistant bacterial transmission across animals or humans by horizontal transfer and clonal spread [11]. Previous studies have shown that antimicrobial-resistant *E. coli* are frequently isolated from dogs and cats [12,13].

The Gram-negative bacterium *E. coli* is a common member of the microbiota of the lower intestine of mammals and, to a lesser extent, birds. *E. coli* is also considered to be the most important reservoir of resistance genes that may be accountable for treatment failures in both human and veterinary medicine [14,15]. In addition, most antibiotic classes used to treat colibacillosis are shared between veterinary medicine and human medicine in the community, regardless of whether these are first-generation antibiotics or critically important antibiotics [16]. Over the past two decades, there has been a significant number of infections caused by *E. coli* carrying extended-spectrum β-lactamases (ESBLs) [17]. ESBLs are becoming more common because this phenotype is being selected for by the use of and exposure to β-lactams [18]. Infection with ESBL *E. coli* (ESBL-EC) is increasingly associated with overt infections in humans and companion animals worldwide [19,20]. ESBL-EC has been identified in the faeces of healthy cats and dogs in a number of studies [21,22].

Virulence factors are usually expressed proteins encoded by genes located in the chromosome or in plasmids. Morato et al. [23] showed that *Escherichia coli* of companion animals is an important source of zoonotic infection. Therefore, further investigations are needed regarding the distribution of virulence genes of *E. coli* isolates from companion animals and their capacity as reservoirs of antimicrobial resistance genes.

Currently, national monitoring programs solely focus on the prevalence of bacteria in food animals, while there is little investigation of the risk of antibiotic resistance switching between humans and companion animals [24]. The prevalence of ESBL-EC in companion animals has been brought to the attention of the scientific community [25,26]; thus, this study aims to estimate antimicrobial resistance in *E. coli* from companion animals in Shandong, China.

## 2. Results

### 2.1. Isolation and Serologic Characterization of E. coli Strains

**Figure 1:**
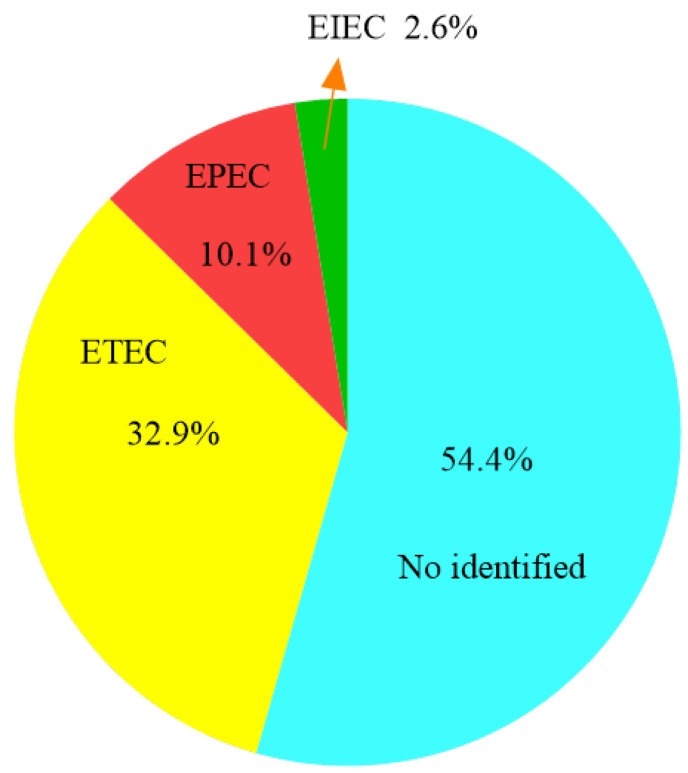
The serotypes (EIEC, EPEC, ETEC and No identified) proportion of *E. coli* isolates from companion animals.

We isolated 79 *E. coli* strains, including 36 (45.6%, 36/79) isolates from apparently healthy companion animals. The *E. coli* isolation rate was 80.6% (79/98). Among these 79 *E. coli* strains, 36 isolates could be serotyped. Furthermore, 36 isolates (45.6%) were found to be DEC in the investigated collection of 79 isolates (Figure 1). Among DEC, ETEC: 26 isolates were found to be the most predominant, and the predominant serotype is O6:K15; followed by EPEC: eight isolates, respectively O125:K70, O128:K67, O114:K90 and O142:K86; EIEC: 2 isolates (Table 1).

### 2.2. Resistance Profiles of E. coli Strains

**Figure 2:**
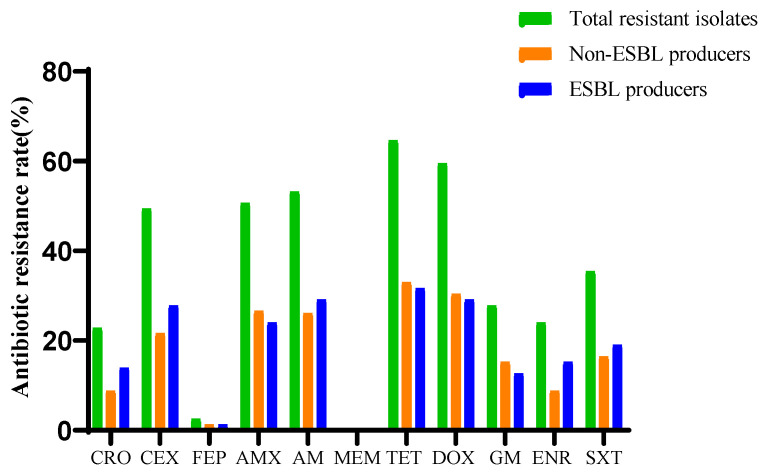
Antimicrobial resistance of ESBL producers and non-ESBL producers.

The results of the antimicrobial susceptibility analysis of 79 *E. coli* isolates are presented in Figure 2. Resistance to TET (64.6%) was the most commonly observed resistance in the *E. coli* isolates. High rates of resistance were also noted for DOX (59.5%) and AM (52.6%). In contrast, all isolates were susceptible to MEM, and a low level of resistance was found for FEP (2.5%). ESBL-EC isolates showed higher levels of resistance to CRO (13.9% vs. 8.9%), CEX (27.8% vs. 21.5%), AM (29.1% vs. 26.1%), ENR (15.2% vs. 8.8%) and SXT (19% vs. 16.4%) than non-ESBL producers. In addition, 49.4% (39/79) of *E. coli* isolates were MDR. Among DEC isolates, higher frequency of MDR strains was detected in ESBL-EC (25%) compared to non-ESBL producing trains (19.4%). On the other hand, resistance to three or more antimicrobial classes was identified in 53.8% and 47.2% of the strains isolated from diarrheic or no-diarrheic companion animals, respectively (Table S1). Noteably, among DEC isolates, all ESBL-EC were observed higher resistance against β-lactames (61.5%), followed by tetracyclines (92.3%) and penicillins (76.9%) (Table 2).

### 2.3. Detection of Antimicrobial Resistance Genes

**Figure 3:**
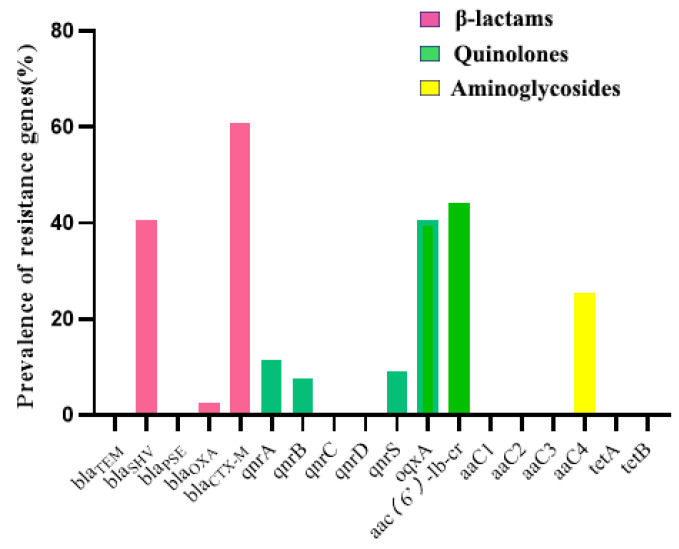
Prevalence of genotypic drug resistance. Resistant genes belonging to the same class of resistant phenotypes are shown in the same color.

All the *E. coli* isolates were analyzed for antimicrobial resistance genes, and the results are shown in Figure 3. Three β-lactamase genes were detected among the isolates, and *bla*_CTX-M_ (60.8%) was the most commonly isolated β-lactamase gene, followed by *bla*_SHV_ (40.5%) and *bla*_OXA_ (2.5%). Five quinolone resistance genes were detected among the isolates, and *aac* *(6’)-Ib-cr* (44.30%) was the most commonly isolated quinolone resistance gene, followed by *oqxA* (40.5%), *qnrS* (19.0%), *qnrA* (11.9%), and *qnrB* (7.6%). Only one aminoglycoside resistance gene, *aaC4* (25.3%) was detected. In addition, we did not detect any tetracycline resistance genes in this study; moreover, *bla*_CTX-M_ was the most prevalent ESBL genotype, and 56.2% (27/48) of *bla*_CTX-M_ positive strains were isolated from healthy companion animals. On the other hand, PMQR genes were found in 79.4% (27/34) ESBL-EC, with 20 isolates harboring β-lactamase genes and PMQR genes (Table S1).

### 2.4. Concordance of Genotypic-Phenotypic Antimicrobial Resistance

The concordance of genotypic and phenotypic resistance is summarized in Table 3. We explored the correlation of genotypic-phenotypic antimicrobial resistance of the isolates. Interestingly, a comparatively stronger correlation was found between ceftriaxone and *bla*_CTX-M_ among the strains (*p*  = 0.010). Additionally, a significant correlation was also observed between phenotypic cephalexin resistance and *bla*_OXA_ gene among isolates (*p*  =  0.007). On the other hand, no statistically significant difference was observed between gentamicin phenotype and *aaC4* resistance gene among strains (*p* >  0.05). On the other hand, all 61 tetracyclines resistant isolates did not harbor *tetA* and *tetB* genes encoding tetracycline resistance.

### 2.5. Distribution of Virulence Genes in E. coli Isolates

**Figure 4:**
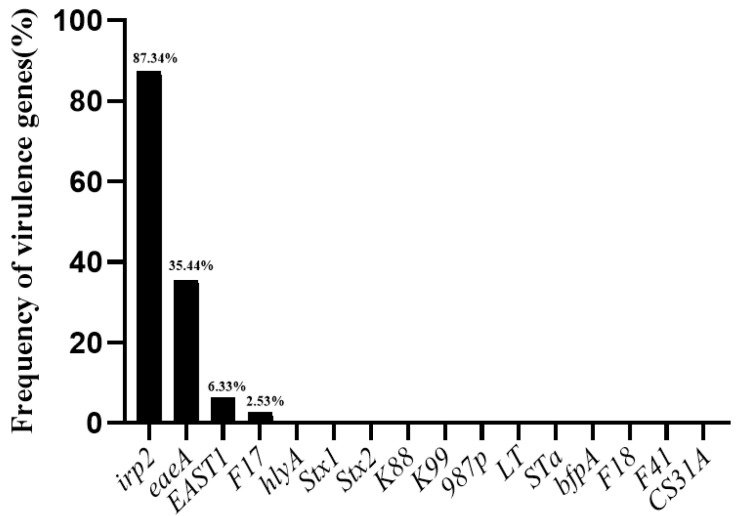
Distribution of virulence genes in *E. coli* strains.

The identification of 16 virulence genes in *E. coli* strains is presented in Figure 4. One or more virulence genes were detected in 93.7% *E. coli* strains. The most prevalent virulence gene was *irp2* (87.3%), the marker gene of high-pathogenicity island, followed by *eaeA* (intimin) (35.4%), *EAST1* (enterotoxins) (6.3%) and *F17* (adhesin) (2.5%). For 34 ESBL-EC isolates, the most prevalent virulence genes were *irp2* (85.3%) and *eaeA* (35.3%). In addition, among DEC isolates, the most frequent virulence genes we observed were *irp2* (91.7%) and *eaeA* (38.9%) is summarized in Table 2. Genes coding for *hlyA*, *Stx1*, *Stx2*, *K88*, *K99*, *987p*, *LT*, *STa*, *bfpA*, *F18*, *F41* and *CS31A* were not detected in any strain. Of these 79 strains, 4 isolates (5.1%) simultaneously carried 3 virulence genes with 2 of them being ESBL-EC, 22 isolates (27.8%) harbored 2 virulence genes, and 5 isolates 5 strains do not carry any of the 11 virulence genes; it is noted that 97.9% (47/48) of isolates from healthy companion animals carried at least one or more virulence genes.

## 3. Discussion

The relationship between companion animals and humans has become increasingly close in recent decades, leading to a high risk of zoonotic transmission of bacteria between pets and humans [27,28,29]. In this study, companion animals were evaluated as a potential source in the transmission of pathogenic bacteria.

Specifically, a total of 79 *E. coli* isolates were confirmed from 98 companion animals (80.6%). Companion animals have been reported as a pivotal transmission reservoir for DEC [30,31]. The findings showed that 36 of 79 strains belonged to DEC, and ETEC was found to be mostly associated with diarrhea companion animals. In previous study, Zahraei et al. [32] found EPEC isolates from 113 non-diarrheic and diarrheic animals. Similar findings were also observed in our study (Table 1), implying that, whether healthy or diarrheic, companion animals may act as a potential reservoir of EPEC. Compared to old animals, young companion animals are usually more susceptible to ETEC and EPEC infection [4]; however, 52.8% (19/36) of pets investigated in our study were older than 12 months. Accordingly, determining the virulence factor, and antibacterial resistance and evaluating the risk of potential transmission to people of companion animals *E. coli* strains are very crucial.

There is concern that antimicrobial resistance in *E. coli* harbored by companion animals can be transmitted from one host to another even by low bacterial numbers [33,34]. Previous studies point out that the close relationship of humans and their companion animals provide opportunities for sharing strains [35,36]. In this study, most of the isolates were resistant to TET (64.6%), DOX (59.5%) and AM (53.2%), which was similar to reports of *E. coli* isolates from other studies [37]. In contrast, other recent studies of *E. coli* isolates from dogs and cats reported resistance to a wide range of antibiotics, including quinolones and extended-spectrum beta-lactamases [38,39]; these high resistance rates are due to the wide use of antibiotics in companion animals. In addition, all the isolates were susceptible to MEM, and the restricted use of carbapenems may be a factor affecting the associated high antimicrobial sensitivity. Carbapenems should be administered only in cases of multidrug-resistant bacterial infection; their restricted use is probably a factor influencing the associated high antimicrobial sensitivity [40,41]. In the current study, the overall MDR frequency of 49.4% was similar to that in studies from the United States (52.0%) and Poland (66.8%) [42,43]. In contrast, the result in this study was much higher than that (28.0%) from healthy dogs from Canada [44]. The high MDR rates observed in the current study indicated that currently available antimicrobial treatment options for *E. coli* infections in companion animals are limited, and it is highly recommended that measures should be taken to control the potential risk from companion animals, such as reducing and standardizing antibiotic usage in clinics.

ESBLs are mainly associated with *E. coli*, which often show resistance to multiple antibiotics [45]. In this study, we found that 43.0% (34/79) of *E. coli* isolates from companion animals produced ESBLs; this result is significantly higher than that reported in Brazil, in which the prevalence of ESBL-EC in companion animals was 8.1% [46]. Most ESBL-EC strains (*n* = 20, 20/34) exhibited the MDR phenotype; this finding may be mirroring the result of irrational use of these antibiotics in veterinary clinics that eventually might cause high selection pressure of resistant bacteria. In Japan, the percentage of ESBL-EC in companion animals was 21.3%, and most of the isolates were sampled from urinary tract infections [47], and it is a common clinical diagnosis that urinary tract infection due to *E. coli* strains. In Europe and the United States, the proportions of ESBL carriers in companion animals varied according to the country and the sampling, but remained under 5% [25,48,49]. Data on the prevalence of ESBL-EC in different studies were difficult to compare based on differences in regions, sample types, and animal health status.

As reported previously, *bla*_CTX-M_ genes are the most frequent ESBL-encoding genes identified in both humans and animals [50,51]. In this study, the prevalence of *bla*_CTX-M_ was 60.8%, which was much higher than that in other studies in which the prevalence of *bla*_CTX-M_ among bacteria derived from pets in different countries (including China) ranged from 10 to 21% [38,52,53]. Furthermore, higher resistance to quinolone antibiotics was observed in ESBL-EC strains, which may be because plasmids containing *bla*_CTX-M_ often carry genes that confer resistance to other antibiotic families [54]. Therefore, the risk of zoonotic transmission of ESBL-EC is likely to be high, and the *E. coli* isolates from companion animals are reservoirs of ESBLs. In addition, we did not find *bla*_TEM_ in this study, which was different from the *E. coli* from food-producing animals in which *bla*_TEM_ was the most commonly identified β-lactamase gene [55].

PMQR is a threat to veterinary clinical therapy. To date, at least three types of quinolone resistance determinants, including the qnr family (*qnrA*, *qnrB*, *qnrS*, *qnrC*, *aac (6’)-Ib-cr* and *qnrD*), and quinolone efflux pumps (*oqxA*), have been extensively reported [56]. In this study, we found a high prevalence of *aac (6′)-Ib-cr* among the *E. coli* isolates, and similar findings have been confirmed among the *E. coli* isolates from companion animals in Australia [57]. In addition, we detected the *qnrS* gene, which was the most prevalent PMQR gene among ESBL-producing *Enterobacter* spp. isolates from humans in China [58] but was not previously detected among isolates from companion animals in Australia [57]; these findings may suggest that the *qnrS* gene is locally spread among companion animals and humans in China.

In our study, 84.4% of non-ESBL producers carried at least 1 or more β-lactamase genes. In addition, 18 isolates of non-ESBL producers were resistant to cephalosporin antibiotics. The difference in phenotypic-genotypic cephalosporin resistance, a likely molecular mechanism was the presence of other AmpC β-lactamase genes. *tetA* and *tetB* genes were not detected in not detected in all tetracyclines resistance isolates, possibly because strains contain some other resistance genes, or involve other resistance mechanisms [59].

The high-pathogenetic island marker gene, *irp2* mediates the iron-uptake system of highly pathogenic strains and was associated with *E. coli* virulence [60]. Several reports showed that *irp2* was detected in pathogenic *E. coli* strains from humans [61,62], we observed a very high frequency of *irp2* gene (87.3%). Additionally, *eaeA* (intimin) observed in DEC were found to be more pathogenic in humans and are involved in zoonotic transmission [63], a similar result was found in our study. Therefore, our study indicates that strains harbored *irp2* may bring a risk for human and companion animals health. On the other hand, *easA* gene was mainly detected from diarrhea companion animals (Table S1), this finding showed that *easA* positive strains may are associated with animal diarrhea mechanism.

## 4. Materials and Methods

### 4.1. Sampling Size Determination

The required sample size was determined based on a confidence interval (CI) of 95%, an *E. coli* expected prevalence of 50% and an accepted error of 10%, giving a value of *N* will be 98 samples in this study according to a previous report [64].

*N* = *Z*^2^α*p*(1 − *p*)/*L*^2^, (1)

where *N* = number of samples, *Zα* = (1 − α/2) percentile of a standard normal, *p* = prevalence, *L* = margin error of 5%.

### 4.2. Bacterial Isolates

Rectal and oronasal swabs were collected from 65 dogs and 33 cats attended in different veterinary clinics in the city of Tai’an, Shandong during a 3-month period (January to March 2021). Specimens were refrigerated until processing. The health status and age of the companion animals are shown in Table S1. Ethical approval was not required for the study because the sampling process did not harm the animals.

The swab samples were tested for the presence of *E. coli* as previously described [65]. The swab samples were streaked onto MacConkey (Hope, Qingdao, China) agar plates and incubated overnight at 37 °C. After overnight incubation, the phenotypic characteristics of *E. coli* were selected, and isolates were subcultured onto MacConkey agar plates. The subcultured colonies were confirmed as *E. coli* by matrix-assisted laser desorption ionization-time of flight mass spectrometry (MALDI-TOF MS) with a Vitek-MS (bioMerieux, Marcy-Etoile, France). Isolates were inoculated into 5 mL of lysogeny broth (LB) and incubated at 37 °C with shaking for 18 h. A 1 mL aliquot of this suspension was combined with 0.5 mL of sterile glycerol and stored at −80 °C. Additionally, *Escherichia coli* ATCC 25,922 was kept in the Laboratory of Veterinary Public Health, Shandong Agricultural University.

### 4.3. Determination of Serotypes, and Genomic DNA Extraction

According to the manufacturer’s instructions (Tianrun, Ningbo, China), using polyvalent and monovalent agglutination sera to serotype. Briefly, commercially available antisera were blended with a *E. coli* suspension on a slide, and then the serotype was determined in one minute.

The isolates were cultivated on 2 mL of LB and incubated at 37 °C with shaking for 10 h. Genomic DNA was extracted by using the Genomic DNA Purification Kit (Tiangen, Beijing, China), and DNA templates were stored at −20 °C until use.

### 4.4. Antimicrobial Susceptibility Testing and Detection of ESBL-EC

All *E. coli* isolates were subjected to antimicrobial susceptibility testing by the broth diffusion method according to the Clinical and Laboratory Standards Institute (CLSI) [66]. The 11 antibiotics tested were ampicillin (AM, 10 μg), amoxicillin (AMX, 10 μg), trimethoprim-sulfamethoxazole (SXT, 25 μg), tetracycline (TET, 30 μg), gentamicin (GM, 10 μg), meropenem (MEM, 10 μg), enrofloxacin (ENR, 15 μg), ceftriaxone (CRO, 30 μg), doxycycline (DOX, 30 μg), cephalexin (CEX, 30 μg) and cefepime (FEP, 30 μg). *E. coli* isolates resistant to more than three classes of antimicrobials were defined as MDR isolates [67]. In addition, the phenotypic evaluation of the ESBL-producing isolates was confirmed by double-disk synergy test according to the guidelines of the CLSI. *E. coli* ATCC 25,922 was used as a quality control strain.

### 4.5. Detection of Resistance Genes

PCR screening was applied for β-lactamase-encoding genes (*bla*_SHV_, *bla*_OXA_, *bla*_CTX-M_, *bla*_TEM_, and *bla*_PSE_), other genes associated with resistance to aminoglycosides (*aaC1*, *aaC2*, *aaC3*, and *aaC4*), plasmid-mediated quinolone resistance genes (*qnrA*, *qnrB*, *qnrC*, *qnrD*, *qnrS*, *oqxA*, *aac (6′)-Ib-cr),* tetracyclines (*tetA and tetB*). All the primers and annealing temperatures were based on slight modifications of those from previously described procedures [68,69,70,71]. Primer sequences and annealing temperature were summarized in Appendix A Table A1. PCR products were separated and visualized on 1.5% agarose gels using ethidium bromide staining. Furthermore, all PCR amplicons were sequenced to confirm gene identity.

### 4.6. Detection of Virulence Genes

The 16 virulence genes encoding adhesin (*K88*, *K99*, *F17*, *F18*, *F41*, *987p*, *CS31A*), bundle-forming pilus (*bfpA*), shiga toxins (*Stx1*, *Stx2*), α-haemolysin (*hlyA*), enterotoxins (*LT*, *Sta*, *EAST1*), yersiniatbactin biosynthesis (*irp2*), and intimin (*eaeA*) of *E. coli* were detected with the primers in Appendix A Table A2 and reaction system previously studies [72,73,74,75].

### 4.7. Statistical Analysis

The statistical analyses were performed using SPSS software version 21.0 (IBM Corp., Armonk, NY, USA), employing the chi-square test. A *p* value of < 0.05 was considered to be statistically significant.

## 5. Conclusions

Overall, our results highlight the seriousness of the antibiotic resistance problem among *E. coli* isolates from whether healthy or diarrhea companion animals in Shandong, China, which may provide a significant reference for pet clinical veterinarians, public health agencies and other researchers. Our findings also emphasize that the presence of ESBL-producing *E. coli* among companion animals needs further continued surveillance. Therefore, it is necessary to establish national standards for the rational use of antibiotics in companion animals.

## Figures and Tables

**Table 1:** Serotypes of DEC *E. coli* isolated from healthy and diarrhea companion animals.

Clinical Status	ETEC (*n* = 26)	EPEC (*n* = 8)	EIEC (*n* = 2)
healthy	O6:K15(4), O78:K80(2), O25:K19(3), O8:K40(2), O9:K9(1), O20:K17(1), O7:K1(1)	O114:K90(1), O128:K67(1), O125:K70(2),	O29:K?(1), O124:K7(1)
diarrhea	O6:K15(4), O78:K80(2), O25:K19(2), O8:K40(3), O15:K?(1)	O114:K90(2), O128:K67(1), O142:K86(1)	-

**Table 2:** Six antibiotic classes resistance of ESBL-EC and non-ESBL producers among DEC isolates.

Antibiotic Classes	Number (%) of Resistant Isolates	
	ESBL (+)	ESBL (−)
	(*n* = 13)	(*n* = 23)
β-lactames	8 (61.5)	10 (43.5)
Tetracyclines	12 (92.3)	17 (73.9)
Quinolones	4 (3.1)	2 (8.7)
Penicillins	10 (76.9)	12 (52.2)
Aminoglycosides	4 (30.8)	8 (34.8)
Carbapenems	0	0

**Table 3:** Correlation matrix between resistance phenotype and genotype.

Antibiotics Resistance Phenotype	Characteristics of Strains					*p* Value ^5^
	n-Pr ^1^	Resistance Genes	n-Gp ^2^	P+/G− ^3^	P−/G+ ^4^	
CRO	18	*bla*_CTX-M_	48	1	31	0.010 *
		*bla*_SHV_	32	11	25	0.166
		*bla*_OXA_	2	1	1	0.260
CEX	39	*bla*_CTX-M_	48	3	12	0.128
		*bla*_SHV_	32	25	18	0.738
		*bla*_OXA_	2	0	2	0.007 **
FEP	2	*bla*_CTX-M_	48	0	46	0.496
		*bla*_SHV_	32	1	31	1.000
		*bla*_OXA_	2	1	1	1.000
GM	22	*aaC4*	20	13	11	0.889
	19	*qnrA*	9	5	4	0.786
		*qnrB*	6	2	4	0.067
ENR		*qnrS*	15	7	3	0.665
		*oqxA*	32	9	22	0.446
		*aac (6′)-Ib-cr*	35	8	24	0.325

^1^ n-Pr: number of strains expressing phenotypic antimicrobial resistance to the indicated antimicrobials. ^2^ n-Gp: number of strains harboring the indicated antimicrobial resistance genes. ^3^ P+/G−: number of phenotypic resistance strains (P+) with no resistance gene (G−) for the antimicrobial identified. ^4^ P−/G+: number of phenotypic susceptible strains (P−) with resistance genes (G+) for antimicrobials. ^5^ *p* value: * *p* < 0.05; ** *p* < 0.01.

## Data Availability

Not applicable.

## Supplementary Materials

The following supporting information can be downloaded at: https://www.mdpi.com/article/10.3390/antibiotics11060828/s1, Table S1: Characteristics of the *E. coli* collected from companion animals.

## Appendix A

**Table A1:** Primers used for resistance genes.

Target Genes	Sequence (5′ → 3′)	Product Size (bp)	Reference
**β-Lactamases**			
*bla*_TEM_	F: ATAAAATTCTTGAAGACGAAA	643	[69]
	R: GACAGTTACCAATGCTTAATC		
*bla*_SHV_	F: TTATCTCCCTGTTAGCCACC	860	[69]
	R: GATTTGCTGATTTCGCTCGG		
*bla*_PSE_	F: TAGGTGTTTCCGTTCTTG	150	[70]
	R: TCATTTCGCTCTTCCATT		
*bla*_OXA_	F TCAACTTTCAAGATCGCA	591	[69]
	R: GTGTGTTTAGAATGGTGA		
*bla*_CTX-M_	F: CGCTTTGCGATGTGCAG	550	[69]
	R: ACCGCGATATCGTTGGT		
**quinolones**			
*qnrA*	F: ATTTCTCACGCCAGGATTTG	519	[69]
	R: GATCGGCAAAGGTCAGGTCA		
*qnrB*	F: GATCGTGAAAGCCAGAAAGG	513	[69]
	R: ACGATGCCTGGTAGTTGTCC		
*qnrC*	F: GGTTGTACATTTATTGAATC	666	[71]
	R: TCCACTTTACGAGGTTCT		
*qnrD*	F: AGATCAATTTACGGGGAATA	984	[71]
	R: AACAAGCTGAAGCGCCTG		
*qnrS*	F: ACGACATTCGTCAACTGCAA	417	[69]
	R: TAAATTGGCACCCTGTAGGC		
*qqxA*	F: GATCAGTCAGTGGGATAGTTT	670	[71]
	R: TACTCGGCGTTAACTGATTA		
*Aac(6’)-Ib-cr*	F: TTGCGATGCTCTATGAGTGGCTA	482	[69]
	R: CTCGAATGCCTGGCGTGTTT		
**aminoglycosides**			
*aaC1*	F: ACCTACTCCCAACATCAGCC	528	[72]
	R: ATATAGATCTCACTACGCGC		
*aaC2*	F: ACTGTGATGGGATACGCGTC	482	[72]
	R: CTCCGTCAGCGTTTCAGCTA		
*aaC3*	F: CACAAGAACGTGGTCCGCTA	185	[72]
	R: AACAGGTAAGCATCCGCATC		
*aaC4*	F: CTTCAGGATGGCAAGTTGGT	286	[72]
	R: TCATCTCGTTCTCCGCTCAT		
**tetracyclines**			
*tetA*	F: GCGCCTTTCCTTTGGGTTCT	211	[72]
	R: CCACCCGTTCCACGTTGTTA		
*tetB*	F: CATTAATAGGCGCATCGCTG	391	[72]
	R: TGAAGGTCATCGATAGCAGG		
	R: AAGCAGACTTGACCTGA		

**Table A2:** Characterization of virulence gene primers used in PCR reactions.

Virulence Factors	Sequence (5′-3′)	Amplicon Size (bp)	Reference
*K88*	F: GATGAAAAAGACTCTGATTGCA	841	[73]
	R: GATTGCTACGTTCAGCGGAGCG		
*K99*	F: CTGAAAAAAACACTGCTAGCTATT	543	[73]
	R: CATATAAGTGACTAAGAAGGATGC		
*F17*	F: GCAGAAAATTCAATTTATCCTTGG	537	[74]
	R: CTGATAAGCGATGGTGTAATTAAC		
*F18*	F: ATGAAAAGACTAGTGTTTATTTCTT	520	[73]
	R: TTACTTGTAAGTAACCGCGTAAGCC		
*F41*	F: GATGAAAAAGACTCTGATTGCA	682	[73]
	R: TCTGAGGTCATCCCAATTGTGG		
*987p*	F: GTTACTGCCAGTCTATGCCAAGTG	463	[73]
	R: TCGGTGTACCTGCTGAACGAATAG		
*CS31A*	F: GGGCGCTCTCTCCTTCAAC	402	[74]
	R: CGCCCTAATTGCTGGCGAC		
*bfpA*	F: GGTCTGTCTTTGATTGAATC	485	[75]
	R: TTTACATGCAGTTGCCGCTT		
*Stx1*	F: ATTCGCTGAATGTCATTCGCT	664	[73]
	R: ACGCTTCCCAGAATTGCATTA		
*Stx2*	F: GAATGAAGAAGATGTTTATAGCGG	281	[73]
	R: GGTTATGCCTCAGTCATTATTAA		
*hlyA*	F: GCATCATCAAGCGTACGTTCC	533	[73]
	R: AATGAGCCAAGCTGGTTAAGCT		
*LT*	F: CCGAATTCTGTTATATATGTC	696	[75]
	R: GGCGACAGATTATACCGTGC		
*Sta*	F: GGGTTGGCAATTTTTATTTCTGTA	183	[73]
	R: ATTACAACAAAGTTCACAGCAGTA		
*EAST1*	F: ATGCCATCAACACAGTATATC	117	[73]
	R: TCAGGTCGCGAGTGACGG		
*irp2*	F: AAGGATTCGCTGTTACCGGAC	287	[73]
	R: TCGTCGGGCAGCGTTTCTTCT		
*eaeA*	F: CATTGATCAGGATTTTTCTGGT	510	[75]
