# Supplementary material for: Companion Animals as Potential Reservoirs of Antibiotic Resistant Diarrheagenic Escherichia coli in Shandong, China
Source: Antibiotics (Basel). 2022 Jun 20;11(6):828. doi: 10.3390/antibiotics11060828 (PMC9220070; doi:10.3390/antibiotics11060828)
Supplement: Supplementary file 1 [file antibiotics-11-00828-s001.zip › antibiotics-1739378-supplementary.pdf]

Supplementary Materials:

Table S1: Characteristics of the *E. coli* collected from companion animals

Isolate	Animal	Age/ month	Clinical status	Serotype1	DEC	ESBL phenotype	Drug resistance spectrum	Resistance genes	Virulence genes
31-2	dog	2	diarrhea	NI	non-DEC	+		bla_{CTX-M}	*eaeA, irp2*
L86-1	cat	2	diarrhea	NI	non-DEC	+	SXT-TET-DOX-AM-AMX-CRO-CEX	bla_{CTX-M}, *oqxA*	*eaeA, irp2*
12-3	cat	36	diarrhea	NI	non-DEC	+	DOX	bla_{SHV}, bla_{CTX-M}, *aac(6')-Ib-cr*	*irp2*
78-1	dog	2	diarrhea	O114:K90	EPEC	+	SXT-TET-DOX-AM-AMX-CRO-CEX	bla_{CTX-M}, *aac(6')-Ib-cr, oqxA, qnrS*	*eaeA, EAST1, irp2*
14-1	cat	24	healthy	NI	non-DEC	+	SXT-TET-DOX-GM-AM-AMX-ENR-CRO-CEX	bla_{SHV}, bla_{CTX-M}, *aaC4, aac (6')-Ib-cr, qnrA, qnrB*	*eaeA*
33-2	cat	2	healthy	O128:K67	EPEC	+	AM-AMX-CRO-CEX	bla_{SHV}, bla_{CTX-M}, *aac(6')-Ib-cr*	*eaeA, irp2*
17-1	cat	12	diarrhea	NI	non-DEC	+	TET-CEX	bla_{SHV}	*irp2*
18	cat	12	diarrhea	NI	non-DEC	+	DOX-GM-AM-AMX-CEX	bla_{CTX-M}, *aaC4*	*eaeA, irp2*
Y1-1	dog	16	diarrhea	NI	non-DEC	+	SXT-TET-DOX-AM-AMX-ENR-CRO-CEX	bla_{CTX-M}, *oqxA, qnrS*	
Y35-1	dog	4	diarrhea	NI	non-DEC	+	SXT-TET-DOX-GM-AM-AMX-ENR-CRO-CEX	*bla*CTX-M, *aaC4, aac(6')-Ib-cr, oqxA, qnrA, qnrS*	*eaeA, irp2*
Y6-3	dog	6	diarrhea	NI	non-DEC	+	SXT-TET-DOX-AM-AMX	*qnrS*	*irp2*

Y58-1	cat	36	diarrhea	O142:K86	EPEC	+	SXT-TET-DOX-AM-AMX-CRO-CEX	*bla*CTX-M, *aaC4*	*irp2*
Y13-1	dog	44	diarrhea	NI	non-DEC	+			
Y45-1	cat	43	diarrhea	O128:K67	EPEC	+	SXT-TET-DOX-AM-AMX-ENR-CRO-CEX	*bla*CTX-M	*irp2*
Y28-1	cat	10	diarrhea	O8:K40	ETEC	+	SXT-TET-DOX-GM-AM-AMX-ENR-CEX	*oqxA*	*irp2*
Y70-3	dog	36	healthy	NI	non-DEC	+	TET-DOX-CEX	*aac(6')-Ib-cr*, *qnrB*	*irp2*
Y102-1	dog	36	healthy	NI	non-DEC	+	TET-DOX-AM-AMX-CEX	*bla*CTX-M, *aac(6')-Ib-cr*	*irp2*
Y94-1	dog	48	healthy	NI	non-DEC	+	TET-GM-AM-AMX-CRO-CEX-FEP	*bla*SHV, *bla*OXA, *bla*CTX-M, *aac(6')-Ib-cr*, *oqxA*, *qnrB*, *qnrS*	*irp2*
Y85-2	dog	60	healthy	O9:K9	ETEC	+	TET-GM-AM-AMX-CEX	*bla*CTX-M, *aaC4*	*irp2*
Y90-1	dog	36	healthy	NI	non-DEC	+	AM-AMX	*bla*SHV, *oqxA*	*irp2*, *EAST1*
Y95-1	dog	120	healthy	NI	non-DEC	+	CEX		*EAST1*
Y99-1	dog	24	healthy	O25:K19	ETEC	+	TET-DOX	*bla*SHV, *bla*CTX-M, *oqxA*	*irp2*
Y105-2	dog	48	healthy	NI	non-DEC	+	SXT-TET-DOX-AM-AMX-ENR-CEX	*bla*CTX-M, *aaC4*, *aac(6')-Ib-cr*, *qnrS*	*irp2*
Y101-1	dog	48	healthy	NI	non-DEC	+			*irp2*
Y98-4	dog	12	healthy	O7:K1	ETEC	+	TET-DOX-GM-AM	*aaC4*, *qnrA*	*irp2*
Y82-1	dog	6	healthy	NI	non-DEC	+		*bla*SHV, *bla*CTX-M, *aac(6')-Ib-cr*, *oqxA*	*irp2*

Y88-2	dog	24	healthy	O25:K19	ETEC	+	SXT-TET-DOX-AM-ENR	*bla*$_{\text{CTX-M}}$, *aaC4*, *aac(6')-Ib-cr*, *qnrA*	*eaeA*, *irp2*
Y64-2	dog	36	healthy	O29:K?	EIEC	+	TET-AM-CEX	*bla*$_{\text{CTX-M}}$, *aaC4*, *aac(6')-Ib-cr*, *qnrA*	*eaeA*, *irp2*
Y41-1	dog	48	healthy	NI	non-DEC	+	SXT-TET-DOX-GM-AM-AMX-ENR-CRO-CEX	*bla*$_{\text{CTX-M}}$, *aaC4*, *aac(6')-Ib-cr*, *qnrA*	*irp2*
Y54-1	cat	12	healthy	O78:K80	ETEC	+	SXT-TET-DOX-GM-AM-ENR-CEX	*bla*$_{\text{OXA}}$, *aaC4*, *aac(6')-Ib-cr*, *oqxA*, *qnrS*	*eaeA*, *irp2*, *EAST1*
Y43-1	cat	10	diarrhea	NI		+	SXT-TET-DOX-AM-AMX-ENR-CRO-CEX	*oqxA*, *qnrS*	*irp2*
Y27-1	cat	10	diarrhea	O8:K40	ETEC	+	TET-DOX	*bla*$_{\text{SHV}}$	
Y33-1	dog	48	healthy	NI		+	TET-DOX-CEX	*aac (6')-Ib-cr*, *oqxA*	*eaeA*, *irp2*
Y24-1	dog	3	healthy	O124:K72	EIEC	+	TET-DOX	*oqxA*	*eaeA*, *irp2*
Y81-2	dog	72	healthy	NI	non-DEC	-	SXT-TET-AM-AMX-ENR	*bla*$_{\text{SHV}}$, *aac(6')-Ib-cr*, *oqxA*, *qnrS*	*irp2*
Y83-1	dog	36	healthy	NI	non-DEC	-	TET-DOX-AMX-CEX	*bla*$_{\text{SHV}}$, *aac(6')-Ib-cr*, *qnrA*, *qnrB*	*irp2*
Y80-1	cat	60	healthy	NI	non-DEC	-	ENR	*bla*$_{\text{SHV}}$, *bla*$_{\text{CTX-M}}$, *aac(6')-Ib-cr*	*irp2*, *F17*
L91-1	cat	18	diarrhea	O6:K15	ETEC	-	DOX-GM-AMX	*bla*$_{\text{SHV}}$	*irp2*
11-1	cat	6	diarrhea	O78:K80	ETEC	-	TET-AM-AMX	*bla*$_{\text{SHV}}$, *bla*$_{\text{CTX-M}}$, *aaC4*, *aac(6')-Ib-cr*, *oqxA*	*eaeA*, *irp2*
71-5	dog	2	diarrhea	NI	non-DEC	-		*bla*$_{\text{SHV}}$, *aaC4*,	*eaeA*, *irp2*

118-2	dog	24	diarrhea	O25:K19	ETEC	-	SXT-TET-DOX	*aac(6′)-Ib-cr, oqxA* *bla*SHV, *aaC4, aac(6′)-Ib-cr, qnrA*	*eaeA, irp2*
16-2	dog	11	diarrhea	NI	non-DEC	-	DOX		
20-1	cat	36	healthy	NI	non-DEC	-	DOX-CEX	*bla*CTX-M, *oqxA, qnrS*	*irp2*
15	dog	2	diarrhea	NI	non-DEC	-	DOX-GM-AM-AMX-CRO-CEX	*bla*CTX-M, *aac(6′)-Ib-cr*	*eaeA, irp2*
10	cat	48	diarrhea	O114:K90	EPEC	-	SXT-TET-GM-AM-AMX-CRO-CEX	*bla*SHV, *bla*CTX-M, *aac(6′)-Ib-cr, qnrB*	*eaeA*
6	cat	18	healthy	NI	non-DEC	-	SXT-TET-AM-AMX-ENR-CEX	*bla*SHV	*irp2*
13	cat	30	healthy	NI	non-DEC	-	GM-AM-AMX-CRO-CEX	*bla*SHV, *bla*CTX-M	*eaeA*
4	cat	42	healthy	NI	non-DEC	-	AM-AMX-CEX	*oqxA*	*eaeA*
Y3-1	dog	2	diarrhea	O6:K15	ETEC	-		*bla*CTX-M, *aaC4, aac(6′)-Ib-cr*	*eaeA, irp2*
Y8-1	dog	12	diarrhea	NI	non-DEC	-	TET-ENR	*bla*SHV, *bla*CTX-M, *aac(6′)-Ib-cr, qnrB*	*irp2*
Y20-1	dog	48	diarrhea	NI	non-DEC	-	DOX-GM	*bla*CTX-M, *aaC4, aac(6′)-Ib-cr, oqxA, qnrS*	*irp2*
Y40-1	dog	96	diarrhea	O25: K19	ETEC	-	TET-GM-AM-AMX-CEX	*bla*SHV, *bla*CTX-M	*eaeA, irp2*
Y17-1	dog	3	diarrhea	O6:K15	ETEC	-	SXT-TET-DOX-GM-AM-AMX-ENR-CEX	*bla*CTX-M, *aac(6′)-Ib-cr, oqxA, qnrS*	*irp2*

Y31-1	cat	16	healthy	O6:K15	ETEC	-	SXT-TET-DOX-GM-AM-AMX-CEX	*bla*$_{\text{SHV}}$, *bla*$_{\text{CTX-M}}$, *oqxA*	*irp2*
Y12-1	cat	10	diarrhea	O78:K80	ETEC	-	TET	*bla*$_{\text{CTX-M}}$	*irp2*
Y18-1	cat	8	healthy	O78:K80	ETEC	-	AM-AMX	*bla*$_{\text{CTX-M}}$	*irp2*
Y5-2	dog	24	diarrhea	NI	non-DEC	-	TET-AMX		*irp2*
Y37-1	cat	36	healthy	O6:K15	ETEC	-	DOX	*bla*$_{\text{CTX-M}}$, *aac(6')-Ib-cr*, *oqxA*	*eaeA*, *irp2*
Y14-1	cat	10	diarrhea	NI	non-DEC	-	TET-AM-AMX-ENR-CRO-FEP	*bla*$_{\text{CTX-M}}$	*irp2*
Y22-2	dog	168	diarrhea	NI	non-DEC	-	AMX		*eaeA*, *irp2*
Y9-1	cat	12	diarrhea	O8:K40	ETEC	-		*aac(6')-Ib-cr*	*eaeA*, *irp2*
Y16-1	cat	16	diarrhea	NI	non-DEC	-	SXT-TET-DOX-GM-AM-AMX-ENR-CRO-CEX	*bla*$_{\text{SHV}}$, *bla*$_{\text{CTX-M}}$, *oqxA*	*eaeA*, *EAST1*, *irp2*
Y65-2	dog	36	healthy	NI	non-DEC	-	TET-DOX-AM-AMX	*bla*$_{\text{CTX-M}}$	*irp2*
Y79-1	dog	24	healthy	O20:K17	ETEC	-	TET-AM-CEX	*bl*$_{\text{aCTX-M}}$, *aaC4*, *aac(6')-Ib-cr*, *oqxA*	*irp2*
Y120-1	dog	60	healthy	NI	non-DEC	-	SXT-TET-DOX-GM-AM-AMX-ENR-CEX	*bla*$_{\text{SHV}}$, *bla*$_{\text{CTX-M}}$, *oqxA*, *qnrS*	*irp2*
Y76-1	dog	24	healthy	NI	non-DEC	-	TET-DOX	*bla*$_{\text{SHV}}$, *bla*$_{\text{CTX-M}}$	*irp2*
Y112-2	dog	96	healthy	O114:K90	EPEC	-	TET-DOX	*bla*$_{\text{SHV}}$, *oqxA*	*irp2*
Y62-3	dog	6	healthy	O6:K15	ETEC	-	SXT-TET-DOX-GM-AM-AMX	*bla*$_{\text{SHV}}$, *bla*$_{\text{CTX-M}}$, *oqxA*, *qnrS*	*irp2*
Y74-3	dog	24	healthy	O125:K70	EPEC	-	SXT-TET-DOX-AM-AMX	*qnrS*	*irp2*
Y84-1	dog	48	healthy	O25:K19	ETEC	-	SXT-TET-DOX-GM-AM-CEX	*bla*$_{\text{SHV}}$, *oqxA*	
Y114-1	dog	16	healthy	O125:K70	EPEC	-	SXT-TET-DOX-GM-AM-AMX-ENR-CRO-CEX	*bla*$_{\text{SHV}}$, *bla*$_{\text{CTX-M}}$, *aaC4*, *aac(6')-Ib-cr*, *qnrA*	*irp2*

Y73-3	dog	72	healthy	O8:K40	ETEC	-	CRO	*bla*SHV, *bla*CTX-M	*irp2*
Y72-3	dog	12	healthy	NI	non-DEC	-	SXT-TET-DOX	*bla*SHV, *bla*CTX-M, *aac(6')-Ib-cr*, *oqxA*	*eaeA*, *irp2*, *F17*
Y71-3	dog	4	healthy	NI	non-DEC	-	SXT-TET-DOX-CEX	*aaC4*, *aac(6')-Ib-cr*	*irp2*
Y92-1	dog	36	healthy	O6:K15	ETEC	-			*irp2*
Y32-1	dog	168	diarrhea	O15:K?	ETEC	-	DOX	*bla*SHV, *bla*CTX-M, *oqxA*	*irp2*
Y15-1	dog	2	diarrhea	O6:K15	ETEC	-		*bla*CTX-M, *aaC4*, *aac(6')-Ib-cr*	*irp2*
Y23-1	dog	16	healthy	NI	non-DEC	-	TET-DOX	*bla*CTX-M, *aac(6')-Ib-cr*, *oqxA*	*eaeA*, *irp2*
Y60-1	dog	3	healthy	O8:K40	ETEC	-	DOX-CEX	*oqxA*	*eaeA*, *irp2*

[1] NI: no identification; +: ESBL producing isolates; -: non-ESBL producing isolates
